# The impact of housing payment problems on health status during economic recession: A comparative analysis of longitudinal EU SILC data of 27 European states, 2008–2010

**DOI:** 10.1016/j.ssmph.2016.05.006

**Published:** 2016-05-24

**Authors:** Amy Clair, Rachel Loopstra, Aaron Reeves, Martin McKee, Danny Dorling, David Stuckler

**Affiliations:** a Department of Sociology, University of Oxford, Manor Road Building, Manor Road, Oxford OX1 3UQ, UK; b European Observatory on Health Systems and Policies, London School of Hygiene and Tropical Medicine, 15-17 Tavistock Place, London WC1H 9SH, UK; c School of Geography and the Environment, University of Oxford, Oxford, Oxford University Centre for the Environment, University of Oxford, South Parks Road, Oxford OX1 3QY, UK

**Keywords:** Housing, Arrears, Multi-level modelling, Fixed effects, Tenure, Comparative

## Abstract

Although the recent Great Recession had its origins in the housing sector, the short-term health impact of the housing crisis is not well understood. We used longitudinal data to evaluate the impact of housing payment problems on health status among home-owners and renters in 27 European states. Multi-level and fixed-effects models were applied to a retrospective cohort drawn from the EU Statistics on Income and Living Conditions survey of employed persons, comprising those without housing arrears in the base year 2008 and followed through to 2010 (*n*=45,457 persons, 136,371 person-years). Multi-variate models tested the impact of transitioning into housing payment arrears on self-reported health (0-worst to 4-best), adjusting for confounders including age, sex, baseline health, and individual fixed effects. Transitioning into housing arrears was associated with a significant deterioration in the health of renters (−0.09 units, 95% CI −0.05 to −0.13), but not owners (0.00, 95% CI −0.05 to 0.06), after adjusting for individual fixed effects. This effect was independent of and greater than the impact of job loss for the full sample (−0.05, 95% CI −0.002 to −0.09). The magnitude of this association varied across countries; the largest adverse associations were observed for renters in Belgium, Austria, and Italy. There was no observed protective association of differing categories of social protection or of the housing regulatory structure for renters. Women aged 30 and over who rented appeared to have worse self-reported health when transitioning into arrears than other groups. Renters also fared worse in those countries where house prices were escalating. We therefore find that housing payment problems are a significant risk factor for worse-self reported health in persons who are renting their homes. Future research is needed to understand potential sources of health resilience among renters, especially at a time when housing prices are rising in many European states.

## Highlights

• Falling into housing arrears is detrimental to health for renters, but not owners.
• The impact of housing arrears on health varies considerably across countries.
• Similarly, tenure differences in health vary across European countries.
• The health of home owners in Poland suffers considerably if they fall into arrears.
• House price increases exacerbate health effects.

## Introduction

The recent Great Recession had its origins in the U.S. housing market, as growing numbers of people fell into rent or mortgage arrears. However, research on the health effects of the recent crisis has focused primarily upon the consequences of job loss and, to a lesser extent, on homelessness, with relatively little attention paid to impact of housing payment problems (Burgard & Kalousova, 2015). This is perhaps surprising given the importance of housing as a determinant of health (Britten, 1934, Muir Gray, 1978, Pevalin et al., 2008, Thomson and Thomas, 2015, Thomson et al., 2013, Winslow, 1937) and the large number of families who have fallen into rent or mortgage arrears during the recession, estimated to be around 3.5 million people across Europe.

Yet as shown in Table 1, not all countries fared equally during the Great Recession. Some countries, such as Italy, saw a slight decline in arrears, falling from 4.3% in 2008 to 4.2% in 2010, while Greece (among others) experienced a substantial rise, from 5.5% to 10.2% during this same period. These marked differences create a unique opportunity to investigate how housing payment problems influence health.

**Table 1:** Prevalence of housing payment arrears in Europe, 2008 and 2010.

	2008	2010	Change 2008–2010
Iceland	5.5	11.2	5.7
Greece	5.5	10.2	4.7
Slovakia	3.0	6.8	3.8
Latvia	3.2	5.8	2.6
Cyprus	3.4	5.6	2.2
Portugal	2.8	4.8	2.0
Spain	4.5	6.4	1.9
Hungary	3.8	5.6	1.8
Denmark	1.1	2.7	1.6
Estonia	1.1	2.7	1.6
Czech Republic	2.3	3.5	1.2
United Kingdom	3.7	4.8	1.1
Lithuania	0.5	1.3	0.8
Netherlands	2.4	3.1	0.7
EU 27	3.3	4.0	0.7
Sweden	1.7	2.3	0.6
Poland	0.6	1.0	0.4
France	5.8	6.1	0.3
Luxembourg	1.1	1.4	0.3
Finland	4.4	4.7	0.3
Bulgaria	1.5	1.7	0.2
Belgium	3.3	3.4	0.1
Austria	3.9	3.9	0.0
Romania	0.6	0.6	0.0
Italy	4.3	4.2	−0.1
Norway	5.0	4.8	−0.2
Slovenia	2.7	2.4	−0.3
Malta	1.5	1.1	−0.4

Notes: Data from Eurostat (n.d.). The average across the 27 countries was 0.7, as such 13 countries are above average and 13 are below, with the Netherlands reporting the average number of arrears.

Housing arrears is one of the so-called ‘soft’ ways in which housing influences health (Shaw, 2004), especially mental health, alongside the ‘hard’, physical impacts of the infrastructure itself, such as damp, mould, and cold. A growing body of scholarship indicates that people who experience housing insecurity, independent of other financial difficulties, experience declines in mental health (Gili et al., 2012, Keene et al., 2015, Meltzer et al., 2013, Meltzer et al., 2011, Nettleton and Burrows, 1998). In Australia, analysis of the longitudinal HILDA dataset found that those in lower income households who had moved into unaffordable housing experienced a worsening in mental health (Bentley, Baker, Mason, Subramanian, & Kavanagh, 2011), with male renters faring worse (Bentley et al., 2012, Mason et al., 2013). Similarly, research comparing US cities found that foreclosures were associated with greater rates of hospital visits (Currie & Tekin, 2011). A qualitative study in five US cities, which included 14 focus groups of low- and mid-income homeowners facing foreclosure as well as foreclosure avoidance professionals, identified housing payment difficulties as a major trigger of psychological feelings of insecurity and worse mental health (Libman, Fields, & Saegert, 2012). Evidence from the US also suggests that foreclosure and eviction during the housing crisis was associated with increased suicide rates (Fowler et al., 2015, Houle and Light, 2014).

Here we seek to expand this literature by investigating how housing payment arrears are related to people's health in Europe, and, based on previous findings, how this impact varies across countries, tenures and individual characteristics. The relationship between housing payment arrears and health will likely vary according to housing tenure given the different circumstances of renters and owners in many nations. Renters tend to have fewer savings, including a lack of equity associated with home ownership, which may render housing hardship more hazardous (Kemeny, 1978, Lowe, 2004, Mason et al., 2013). Such differences in outcomes by tenure have been identified in other nations including Australia and the USA (Bentley et al., 2011, Bentley et al., 2012, Mason et al., 2013), here we investigate whether the same is true in Europe.

The health impacts of economic shocks such as job loss have been shown to vary across countries dependent on social support and protection, a stark example being the difference between Iceland and Greece's responses and resulting health issues during the Great Recession (Stuckler & Basu, 2013). Housing market characteristics, such as prices and the ownership rate, vary considerably across Europe, as do levels of social protection and informal social support, such as social capital. Here we investigate whether experiencing housing payment problems has different consequences, including health impacts, depending on where a person lives and what differences in social support may best explain this variation.

Both owners and renters risk housing payment problems, with potentially differing consequences for health. Renters are more likely to experience homelessness as a result of arrears, whereas those who have mortgages face the potential additional loss of substantial accumulated capital. However, owners may also have greater resilience, resulting from greater financial resources and housing market options. To our knowledge, while it is very plausible that renters may be more vulnerable than those with a mortgage, few studies have sought to differentiate these groups (Mason et al., 2013, Pollack et al., 2010).

The causes and consequences of transitioning into housing payment arrears are similarly likely to vary according to individual characteristics and life stages. Women, who often still take on the bulk of caring duties, may experience a greater reduction in health when facing payment problems than men, for example, although Australian evidence suggests that men may fare worse and evidence from Britain indicates men are more likely to seek medical support (Nettleton & Burrows, 1998). Older people who fall into arrears may have fewer options available to them, increasing the risk of health consequences. In contrast, younger adults may have fewer resources available, having had less time to accumulate savings for example, but may have parental support.

Using the only source of longitudinal data from 27 European nations which covers both housing and health data, we test the hypotheses that transitioning into housing arrears worsens people's health and that this effect is worse for renters than owners, varies across countries and according to individual characteristics.

## Methods

### Data sources

We use the 2010 longitudinal EU-SILC dataset to construct a retrospective cohort of employed persons who had no housing payment arrears in 2008. Details of EU SILC have been described elsewhere (Arora et al., 2015, Europa, 2015, Iacovou et al., 2012), but briefly it uses a four-year rotating panel sample with 25% of the sample being replaced in each wave. The precise methods of data collection vary across the (then) 27 Member States, although standards are set centrally. Following previous analyses of effects of the Great Recession (Gili et al., 2012, Reeves et al., 2013, Reeves et al., 2015, Stuckler et al., 2012, Stuckler et al., 2009), we selected the 2010 dataset and used 2008 as the baseline year as, empirically, it corresponds with the onset of the recession and associated rise in housing payment arrears across Europe. Selecting this 3 year period means that our analysis can cover the period of the recession while ensuring a decent longitudinal sample size as fewer sample rotations are lost than if a 4 year dataset were used. Only those respondents that participated in all 3 surveys were included, as limiting the sample in this way better enables us to look at the effects of the transition into housing payment arrears including through the use of fixed-effects models, giving us health and arrears information at 3 time points.

Longitudinal data were available for 25 of the then 27 EU member states, the exceptions being Germany and Ireland. In addition, we included data for Iceland and Norway, yielding a total of 27 nations. The overall individual non-response rate (accounting for multiple interviews in the 2010 dataset) varies across countries, from around 10% in Cyprus to over 40%, the maximum non-response permitted by Eurostat, in Denmark and Norway (Eurostat, 2012). EU-SILC requires representative probabilistic samples, which was achieved in all the countries with the exception of Spain, which used substitution methods to replace non-respondents (Arora et al., 2015). Population registers and censuses were used to assess the representativeness of the samples (Eurostat, 2012).

Respondents were selected for analysis on the basis of being aged 16 or over, not living in rent free accommodation and being both employed and not in housing payment arrears in 2008. These restrictions were applied to ensure that the analysis would capture those most affected by the recession rather than those already experiencing difficulty. This resulted in a sample size of 136,371 person/year responses (45,457 individuals) with country sample sizes varying between 294 person/years for Bulgaria and over 14,000 for France and The Netherlands.

Transitioning into housing payment arrears was a dichotomous measure of whether a household entered into arrears across survey waves, as assessed by the following question: “In the last twelve months, has the household been in arrears, i.e. has been unable to pay on time due to financial difficulties for: (a) rent (b) mortgage repayment, for the main dwelling?”

Following convention we assessed self-reported health using a scale from 0 – very bad to 4 – very good (Singh-Manoux et al., 2006). It is well-known that self-reported health is subject to perceptual and cultural biases (Jürges, 2007, Zimmer et al., 2000). However it is a widely used indicator of individual health and correlates strongly with mortality in many countries, provides a summary of overall health problems, and, when measured repeatedly in the same individuals, avoids potential cross-cultural biases (Ganna and Ingelsson, 2015, Jylhä, 2009).

### Statistical models

We use individual fixed effect models to investigate the relationship between housing payment arrears and health, as follows:

(1) $${\mathrm{Health}}_{i,j,k}{=\;\alpha }_{0}{+\;\beta }_{1}{\mathrm{Arrears}}_{i,j,k}{+\;\beta }_{2}{\mathrm{Age}}_{i,j,k}{+\;\beta }_{3}{\mathrm{Income}}_{i,j,k}\;{+\;\beta }_{n}{\mathrm{DEM}}_{i,j,k}{+\gamma }_{i}{+\varepsilon }_{i,j,k}$$

Here *i* is individual, *j* is country, and *k* is year. *γ_i_* represents the individual fixed effects and *ε_i,j,k_* the individual error term. DEM represents a vector of control variables, including marital status, education level, and housing tenure. All models are clustered by country to reflect non-independence of sampling. All regression models were weighted by person weights, to represent populations. Multilevel analysis with household and country level clustering is also conducted. To facilitate interpretation of results we standardised the health outcome for analysis.

In subsequent models we evaluated a range of potential modifying factors using a series of interaction terms. These potential modifiers included country-level macroeconomic risks, social capital and support, and social protection expenditure, as well as age and gender. All models were performed using Stata 13 (xtreg and xtmixed commands). Table 2 provides descriptive statistics for the variables used in the study.

**Table 2:** Descriptive statistics for outcome and predictor variables in retained sample.

		2008 (%)	2009 (%)	2010 (%)	Data source
Individual variables					
Good Health (0 worst-4 best)		3.12	3.09	3.07	EU SILC
Housing payment arrears	No arrears	45 457 (100%)	39 654 (96.3%)	39 991 (96.0%)	
	Arrears	0	1508 (3.66%)	1672 (4.01%)	
Tenure	Owner-occupier	34 226 (75.3%)	34 864 (76.8%)	35 326 (77.8%)	
	Private rent	7967 (17.5%)	7534 (16.6%)	7282 (16.0%)	
	Reduced rent	3256 (7.16%)	3021 (6.65%)	2794 (6.15%)	
Age		41.4	42.4	43.4	
Gender	Male	24 111 (53.0%)	24 111 (53.0%)	24 111 (53.0%)	
	Female	21 346 (47.0%)	21 346 (47.0%)	21 346 (47.0%)	
Marital status	Married	28 613 (63.0%)	29 067 (64.1%)	29 463 (64.9%)	
	Never married	12 607 (27.8%)	11 993 (26.4%)	11 504 (25,4%)	
	Separated/divorced	3594 (7.92%)	3706 (8.17%)	3756 (8.28%)	
	Widowed	579 (1.28%)	607 (1.34%)	654 (1.44%)	
Education level	Primary only	2411 (5.37%)	2359 (5.24%)	2337 (5.19%)	
	Secondary only	26 007 (57.9%)	25 872 (57.5%)	25 840 (57.36%)	
	Post-secondary	16 472 (36.7%)	16 761 (37.3%)	16 874 (37.5%)	
Disposable income (1000 s)		43.7	44.5	44.5	
Chronic illness at baseline	No	29 658 (80.3%)	–	–	
	Yes	7293 (19.7%)	–	–	
Limiting illness at baseline	No	32 393 (87.7%)	–	–	
	Yes	3760 (10.2%)	–	–	
	Yes, strongly limiting	781 (2.1%)	–	–	
Economic activity	Employed	45 457 (100%)	41 637 (92.0%)	40 528 (89.6%)	
	Unemployed	–	1530 (3.38%)	1812 (4.01%)	
	Retired	–	750 (1.66%)	1343 (2.97%)	
	Other inactive	–	1312 (2.90%)	1549 (3.42%)	
Population level variables					
*Housing*					EEE (Eurostat, 2015)
Home-ownership rate		75.6%	73.5%	73.1%	
Change in house prices		2.55	−5.10	1.72	
Arrears rate		3.39%	4.06%	4.47%	
*Macroeconomic*					
Change in GDP		142	−2330	1220	
Interest rate		4.53%	4.23%	3.78%	
*Social spending (Purchasing Power Standard per inhabitant, divided by 100)*					
Disability		5.27	5.11	5.15	
Total welfare		58.27	58.53	59.48	
Old age		22.79	22.99	23.79	
Survival benefits		3.41	3.33	3.37	
Health		16.99	16.66	16.63	
Family and child		5.30	5.15	5.18	
Unemployment		2.54	3.27	3.31	
Housing		0.78	0.79	0.76	
*Social Capital*					(EVS, 2008)
Most people can be trusted		34.31	–	–	
People helpful most of the time		36.78	–	–	

Sample countries: Austria, Belgium, Bulgaria, Cyprus, Czech Republic, Denmark, Estonia, Greece, Spain, Finland, France, Hungary, Iceland, Italy, Lithuania, Luxemburg, Latvia, Malta, The Netherlands, Norway, Poland, Portugal, Romania, Sweden, Slovenia, Slovakia, and the UK.

Housing payment arrears and economic activity other than employed are zero in 2008 due to sample constraints. House price data are missing for Norway and Iceland. Interest rates missing for Norway, Iceland and Estonia. Social capital data missing for Iceland, Spain and the Czech Republic. Rental regulation information is missing for Cyprus, Czech Republic, Estonia, Hungary, Iceland, Malta, Norway, Romania, and Slovenia.

## Results

### Association of transitioning into housing payment arrears and health status

Table 3 shows the results of a series of multi- and single-level statistical models progressively incorporating control variables. As shown in the first column, the unadjusted association of transitioning into housing payment arrears with worse health, controlling for household and country clustering, is −0.10 units (95% CI: −0.07 to −0.12). After adjusting for baseline health, education, and potential confounding factors, the association was slightly attenuated to −0.07 (95% CI: −0.05 to −0.10). Including individual fixed effects yielded the most conservative estimated association of −0.06 (95% CI −0.02 to −0.10).

**Table 3:** Estimated impact of transitioning into housing payment arrears on self-reported health, 2008–2010, baseline sample of individuals not in arrears and employed.

	Full sample			Owners	Renters
Covariate	Unadjusted multilevel	Adjusted multilevel model	Individual Fixed effects	Individual Fixed effects	Individual Fixed effects
Housing payment arrears	−0.10***	−0.07***	−0.06**	0.00	−0.09***
	(0.01)	(0.01)	(0.02)	(0.03)	(0.02)
Age	-	−0.03***	0.02	0.02	0.01
		(0.00)	(0.02)	(0.03)	(0.02)
Age^2^	-	0.00***	0.00	−0.00	−0.00***
		(0.00)	(0.00)	(0.00)	(0.00)
Female	-	−0.04***	-	-	-
		(0.00)			
Marital status					
Married	-	0.01	0.04	−0.01	0.15**
		(0.01)	(0.03)	(0.03)	(0.05)
Separated or divorced	-	−0.01	−0.04	−0.01	−0.05
		(0.01)	(0.04)	(0.04)	(0.05)
Widowed	-	−0.08***	0.10	−0.18	0.37
		(0.02)	(0.12)	(0.10)	(0.21)
Never married	-	Ref.	Ref.	Ref.	Ref.
Education level					
Post-secondary	-	0.21***	0.03	0.03	0.05
		(0.01)	(0.08)	(0.08)	(0.11)
Secondary	-	0.10***	0.02	0.00	0.03
		(0.01)	(0.10)	(0.09)	(0.10)
Primary	-	Ref.	Ref.	Ref.	Ref.
Tenure					
Private rent	-	−0.06***	−0.03	-	-
		(0.01)	(0.02)		
Reduced rent	-	−0.06***	−0.01	-	-
		(0.01)	(0.02)		
Owner occupier		Ref.	Ref.	-	-
Disposable income (1000 s)	-	0.00***	0.00	0.00	0.00
		(0.00)	(0.00)	(0.00)	(0.00)
Chronic Illness at baseline year (yes)	-	−0.41***	-	-	-
		(0.01)			
Limiting illness at baseline year					
Yes, strongly limiting	-	−0.82***	-	-	-
		(0.02)			
Yes	-	−0.41***	-	-	-
		(0.01)			
No	-	Ref.	-	-	-
Economic activity					
Lost job	–	−0.09***	−0.05*	−0.03	−0.04
		(0.01)	(0.02)	(0.03)	(0.05)
Retired	–	−0.05**	0.02	0.05	−0.00
		(0.02)	(0.04)	(0.04)	(0.07)
Other inactive	–	−0.12***	−0.04	−0.03	−0.05
		(0.01)	(0.03)	(0.03)	(0.04)
Employed	–	Ref.	Ref.	Ref.	Ref.
Individual fixed effects	N	N	Y	Y	Y
Country-level variables	N	N	Y	Y	Y
Number of individual-years	103041	101155	91547	65264	26285
Countries in sample	27	27	24	24	24

Note: Renters include those who rent at a reduced rate and market rate. Country-level variables are those population level variables given in Table 2. Multilevel models account for within-individual (time), household and country-level clustering.

* *p*< 0.05.

** *p* < 0.01.

*** *p* < 0.001.

We then disaggregated these associations by owners and renters, proceeding with the individual fixed effects regression models. As shown in Table 3, there was no significant association between housing payment arrears and health among owners (0.00, 95% CI: −0.05 to 0.06), but there was among renters (−0.09, 95% CI: −0.05 to −0.13).

Next we further investigated whether these impacts were different by age and sex, focusing on renters. As shown in Table 4, presenting 12 separate fixed effects models, we observed that associations were greatest among women aged 30 to 59 (−0.17, 95% CI −0.09 to −0.26) and particularly women aged 60 and over (−1.12, 95% CI −1.03 –to −1.21). These results are in contrast to results from Australia, which indicated that male renters fared worse when facing housing payment difficulties (Bentley et al., 2011, Bentley et al., 2012).

**Table 4:** Association of transitioning into housing payment arrears on self-reported health among renters.

Age	Gender		
	Women	Men	All
Under 30	0.08	−0.06	0.00
	(0.05)	(0.08)	(0.05)
30–59	−0.17***	−0.06	−0.11***
	(0.04)	(0.04)	(0.03)
60 and over	−1.12***	−0.31	−0.40
	(0.04)	(0.18)	(0.22)
All	−0.12**	−0.08*	−0.09***
	(0.04)	(0.03)	(0.02)

Notes: Results presented from 12 separate fixed effects statistical models. Robust standard errors in parentheses.

* *p*< 0.05,

** *p* < 0.01,

*** *p*< 0.001.

### Cross-national differences in the impact of housing payment arrears

**Fig. 1:**
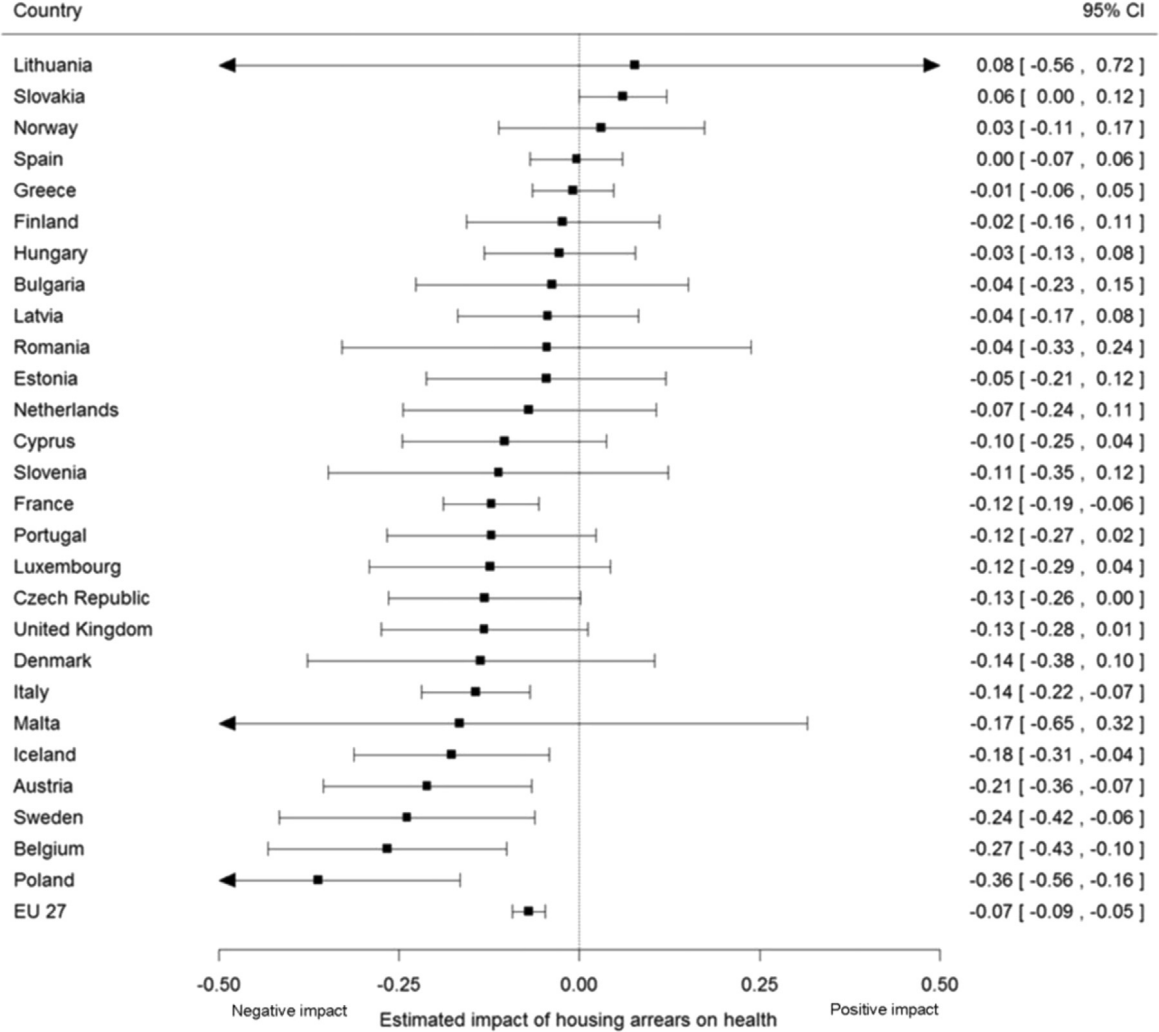
Estimated impact of housing payment arrears on self-reported health with 95% confidence intervals, 2008–2010.

**Fig. 2:**
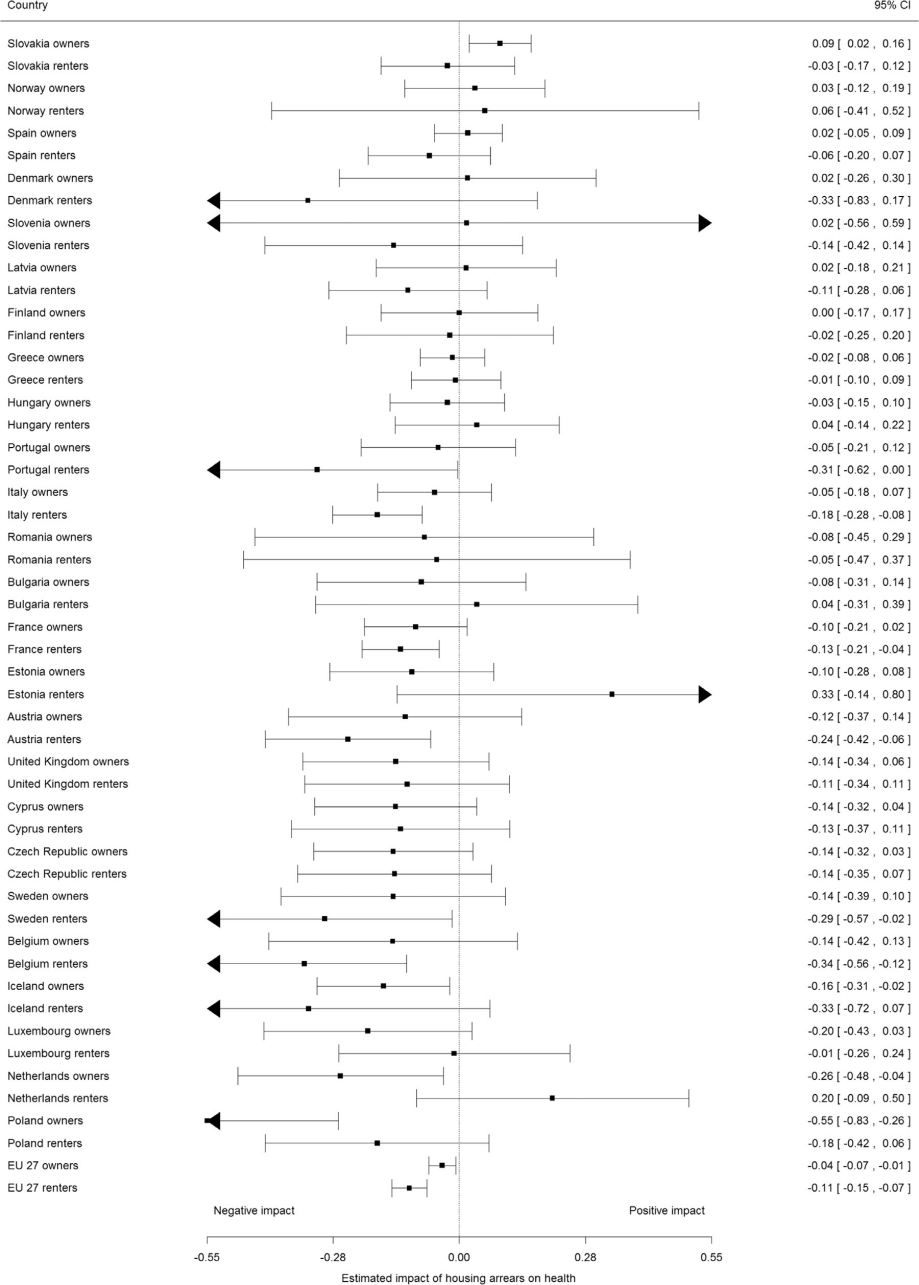
Estimated impact of housing payment arrears on self-reported health with 95% confidence intervals, 2008–2010, by tenure.

Having observed that renters had worse health, we next investigated differing impacts across countries. Fig. 1, Fig. 2 plot the country-specific associations of transitioning into housing payment arrears with self-reported health, both overall (Fig. 1), and disaggregated by tenure (Fig. 2). The estimated impact varied considerably. In some countries, there was no effect of housing payment arrears, while in others, such as Poland, the negative effect of arrears on health is considerably larger than average (−0.36 compared to the average −0.07, both *p*<0.05). Although there are a few exceptions, we observed that in the majority of countries, including those diverse as Belgium, Iceland, Austria, and Sweden, renters are much worse off than owners when facing housing payment arrears.

Next we sought to investigate potential factors which may exacerbate or mitigate this impact, given the marked variation across countries. First we investigated characteristics of the housing market using a series of interaction terms. Table 5 shows the results. We observed that in countries with greater prevalence of homeownership, both owners and renters appeared to have slightly attenuated health risks associated with experiencing housing payment arrears. Additionally, rising housing prices appeared to exacerbate the estimated impact of housing payment arrears on health status.

**Table 5:** Interaction of potential modifying factors with the housing payment arrears-health association, baseline sample of individuals not in arrears and employed, multilevel analysis.

Modifying factor	Owners	Renters
*Macroeconomic*		
Home ownership rate	0.006**	0.006**
	(0.002)	(0.002)
House price change	−0.004*	−0.006*
	(0.002)	(0.003)
GDP change	−0.00	−0.002*
	(0.00)	(0.001)
Interest rate	0.00	0.02*
	(0.01)	(0.01)
Country arrears rate	0.02**	0.02*
	(0.01)	(0.01)
*Social Capital*		
People are helpful most of the time	−0.00	−0.00
	(0.00)	(0.00)
Most people can be trusted	−0.00	−0.00
	(0.00)	(0.00)
*Social protection expenditure*		
Disability spending	−0.01	−0.01
	(0.00)	(0.01)
Total social protection spending	−0.001*	−0.00
	(0.001)	(0.00)
Old age social protection spending	−0.004*	−0.005*
	(0.002)	(0.002)
Survival benefits spending	−0.01	−0.01
	(0.01)	(0.01)
Health spending	−0.005*	−0.00
	(0.002)	(0.00)
Family and child spending	−0.01	−0.01
	(0.00)	(0.01)
Unemployment spending	0.00	−0.01
	(0.01)	(0.01)
Housing spending	−0.05**	−0.01
	(0.02)	(0.02)
*Rental market regulations*		
Rent controls	−0.04	−0.01
	(0.04)	(0.04)
Tenant-landlord relationship	−0.03	−0.02
	(0.04)	(0.04)

Note: Models include all individual level control variables as used in the previous table, separate models run for each potential interaction. Standard errors in parentheses

^***^p<0.001.

* *p*<0 .05.

** *p*< 0.01.

We then tested whether greater rental market regulation was protective, using binary measures taken from a European Commission Study (Cuerpo, Kalantaryan, & Pontuch, 2014). These binary measures capture whether a country has a higher level of tenancy protection, either through rent controls or favourable positioning of tenants in negotiations with landlords. None found a significant interaction with housing payment arrears.

Finally we included a set of formal and informal social support measures. The formal ones included alternative categories of social protection expenditure, including housing support, old-age spending, healthcare, family support and unemployment benefits. The informal ones involved social capital factors, the proportion in a country responding that people are helpful most of the time and whether people can be trusted. None of the many variables tested had a significant protective effect on the housing payment arrears-health status association.

### Robustness checks

Thus far we have treated the health outcomes as a scale variable for ease of interpretation. However treating health in this way may have affected our results. We check this by re-running the models shown in Table 3 using ordinal analyses, the results of which are given in Appendix Table A1. The results of these models are consistent with the linear approach, finding that housing payment arrears is associated with deterioration in health for the full sample, which reflects a large effect for renters and a non-significant effect for owners when disaggregated by tenure.

As a further check we run a number of longitudinal linear probability models where the self-reported health outcome has been transformed into a binary variable (Good/very good health coded as 0, fair/bad/very bad as 1). The results of these models are given in Appendix Table A2. The findings are equivalent to our previous models, finding that housing payment arrears is associated with an increased risk of reporting poor health, which when disaggregated by tenure reflects a large risk for renters and no statistically significant risk for owners.

In order to further investigate the differences found across tenures we investigate whether the differing characteristics of owners and renters may account for some of the variation found. Web Appendix Table A3 gives the descriptives for the sample disaggregated by tenure. As would be expected there are differences in the characteristics of individuals in these different tenures. We ran fixed-effects interaction models (equivalent to the third model in Table 3) for all of the time-varying individual-level predictors to investigate whether any differences in sample characteristics may help us to further understand the health consequences of housing payment arrears. Only one significant interaction was found, indicating that widow(er)s report higher health when living in reduced rate rented homes.

## Discussion

Our analysis demonstrates that transitioning into housing payment arrears is a significant risk factor for worsening health status across Europe. We found that these negative associations were concentrated in renters, especially women aged 30 and over. However, in most countries, a short term period of housing arrears had no identifiable impact on owners’ health status. Escalating house prices appeared to render renters more vulnerable to the health consequences of payment problems.

As with all observational studies, our analysis has several limitations. First, reflecting the availability of comparative data, we focused on the financial aspects of housing. This does not reflect the many aspects of housing difficulties, including low quality or substandard housing. Second, although our analysis utilised the longest series of available comparative longitudinal data for a large number of European countries, it still is limited to a relatively short duration. This makes the effects observed more likely to reflect mental, rather than physical health, many of which would require longer time periods to develop. Further research is also needed to investigate the longer-term consequences and potential scarring effects of housing insecurity. Third, we failed to identify statistically significant protective factors, whether from social protection or social capital. This may reflect a low degree of statistical power when these protective factors are observed at the country level or the crude nature of such measures which do not capture how, as well as how much, money is spent. Given the number of countries included in our study and the complexity and variety that characterises housing policy, it has not been possible to thoroughly investigate the relative protections afforded to owners and renters across countries. Additional studies are needed to understand the reasons for the considerable differences across nations in the risks faced by renters. Fourth, one strength of our study is that we were able to adjust for baseline health and identify changes in persons who experienced new payment problems. While we adjust for baseline health it remains possible that people's health may influence their likelihood of falling to housing payment arrears given the period of time between data collection points. However we believe this unlikely given our analytical approach. The controls for individual fixed effects may also yield conservative estimates of the association of housing with worse health status. Fifth, again reflecting the lack of available comparative data for a large number of EU countries, we were unable to disaggregate those facing housing payment problems of different levels of severity.

Our study points to several directions for future research, which should be a priority given that difficulties in the housing sector are likely to persist in Europe. One is to account for not only demand-side factors, which shape people's ability to afford housing, but also supply-side factors, which may help contain escalating housing costs, such as rent controls and provision of social housing. Further research is needed to develop and use better measures of rental controls and negotiating power of renters, likely necessitating new data collection across Europe.

Several observations of outliers are noteworthy. We observed that, in contrast to the general trend in Europe, owners in Poland had greater risk of health problems than did renters. This may reflect exposure to foreign currency debt, used in some nations to purchase homes. Approximately 60% of Polish mortgages were denominated in foreign currency, often from Swiss banks, exposing them to exchange rate fluctuations that may not have been anticipated (Miles & Pillonca, 2008). In Austria and Belgium renters who experience housing payment arrears appeared to fare particularly poorly compared with owners and the rest of Europe. Such unanticipated shocks, which occur outside the control of individuals, may be particularly deleterious. This is also consistent with our observation that escalating housing prices in a nation appeared to exacerbate the impacts of arrears on health status.

In some European countries, especially parts of the UK, housing prices have risen and are projected to remain high. While policies are in place in some countries to subsidise and support people experiencing arrears, less attention has been paid to intervening directly in the market to render housing more affordable. In some cases, policies designed to facilitate home ownership have perversely increased prices, so worsening the risk to health. For example in the UK (particularly England) official policies have the effect of maintaining or increasing already high house prices, either directly or through encouraging the perception of housing as an asset, with examples including ‘Buy to Let’ and ‘Help to Buy’, although April 2016 tax changes have made the former less attractive for landlords, with as yet unknown consequences.

Overall our results suggest that, similar to job loss, housing payment arrears is a major public health concern especially in times of economic downturn and hardship. As well as seeking to reduce instances of housing payment arrears, research should investigate how to mitigate the impacts of arrears on health, and understand why some groups are more severely affected than others.

## Conflicts of interest

None declared.

## Figures and Tables

**Table A1:** Estimated impact of transitioning into housing payment arrears on self-reported health, 2008–2010, baseline sample of individuals not in arrears and employed, ordinal models.

Covariate	Full sample				Owners	Renters
	Unadjusted multilevel	Adjusted multilevel model		Longitudinal ordinal RE	Longitudinal ordinal RE	Longitudinal ordinal RE
Housing payment arrears	−0.42***	−0.31***		−0.21*	0.02	−0.43***
	(0.06)	(0.05)		(0.09)	(0.10)	(0.08)
Age	–	−0.13***		−0.13***	−0.12***	−0.15***
		(0.01)		(0.02)	(0.02)	(0.02)
Age^2^	–	0.00***		0.00***	0.00*	0.00***
		(0.00)		(0.00)	(0.00)	(0.00)
Female	–	−0.18***		−0.20***	−0.14*	0.32***
		(0.02)		(0.04)	(0.06)	(0.04)
Marital status						
Married	–	0.04	0.10		0.08	0.10
		(0.03)	(0.05)		(0.05)	(0.09)
Separated or divorced	–	−0.02	0.05		0.11	0.01
		(0.05)	(0.07)		(0.09)	(0.11)
Widowed	–	−0.27**	−0.26*		−0.32*	−0.13
		(0.10)	(0.11)		(0.13)	(0.17)
Never married	–	Ref.	Ref.		Ref.	Ref.
Education level						
Post-secondary	–	0.94***		1.17***	1.16***	1.18***
		(0.05)		(0.17)	(0.20)	(0.14)
Secondary	–	0.44***		0.61***	0.63**	0.55***
		(0.05)		(0.17)	(0.19)	(0.14)
Primary	–	Ref.		Ref.	Ref.	Ref.
Tenure						
Private rent	–	−0.27***		−0.12	-	-
		(0.03)		(0.07)		
Reduced rent	–	−0.31***		−0.25**	-	-
		(0.04)		(0.07)		
Owner occupier	–	Ref.		Ref.	-	-
Disposable income (1000 s)	–	0.004***		0.01***	0.00***	0.01**
		(0.000)		(0.00)	(0.00)	(0.00)
Chronic Illness at baseline year (yes)	–	−1.81***		−1.85***	−1.90***	−1.70***
		(0.04)		(0.11)	(0.10)	(0.13)
Limiting illness at baseline year						
Yes, strongly limiting	–	−3.09***		−3.13***	−3.18***	−3.05***
		(0.09)		(0.19)	(0.20)	(0.27)
Yes	–	−1.63***		−1.72***	−1.77***	−1.66***
		(0.05)		(0.09)	(0.09)	(0.14)
No	–	Ref.		Ref.	Ref.	Ref.
Economic activity						
Lost job	–	−0.35***		−0.39***	−0.43**	−0.31**
		(0.05)		(0.10)	(0.14)	(0.12)
Retired	–	−0.18*		−0.16	−0.08	−0.30
		(0.08)		(0.10)	(0.14)	(0.16)
Other inactive	–	−0.40***		−0.39***	−0.40***	−0.35**
		(0.06)		(0.08)	(0.10)	(0.10)
Employed	–	Ref.		Ref.	Ref.	Ref.
Individual fixed effects	N	N		N	N	N
Country-level variables	N	N		Y	Y	Y
Number of individual-years	103041	101155		92931	66026	26907
Countries in sample	27	27		24	24	24

Note: Renters include those who rent at a reduced rate and market rate. Country-level variables are those population level variables given in Table 2. Multilevel models account for within-individual (time), household and country-level clustering. Log odds coefficients. Fixed effects models not available, all models unweighted as weighting not supported

* *p* < 0.05.

** *p* < 0.01.

*** *p* < 0.001.

**Table A2:** Estimated impact of transitioning into housing payment arrears on self-reported health, 2008–2010, baseline sample of individuals not in arrears and employed, longitudinal linear probability models with fixed effects.

Covariate	Full sample	Owners	Renters		
	Unadjusted longitudinal	Adjusted longitudinal	Longitudinal with country vars	Longitudinal with country vars	Longitudinal with country vars
Housing payment arrears	0.05***	0.04**	0.04**	−0.00	0.07***
	(0.01)	(0.01)	(0.01)	(0.01)	(0.01)
Age	–	−0.01	−0.01	−0.01	−0.02
		(0.01)	(0.01)	(0.01)	(0.02)
Age^2^	–	0.00*	0.00*	0.00	0.00
		(0.00)	(0.00)	(0.00)	(0.00)
Female	–	–	–	–	–
Marital status					
Married	–	−0.01*	−0.01	0.01	−0.06**
		(0.01)	(0.01)	(0.01)	(0.01)
Separated or divorced	–	0.04**	0.04**	0.02	0.07*
		(0.01)	(0.01)	(0.02)	(0.03)
Widowed	–	0.01	0.00	0.04	−0.05
		(0.06)	(0.07)	(0.05)	(0.11)
Never married	–	Ref.	Ref.	Ref.	Ref.
Education level					
Post-secondary	–	−0.03	−0.02	0.02	−0.09
		(0.04)	(0.04)	(0.04)	(0.05)
Secondary	–	0.00	−0.01	0.02	−0.05
		(0.01)	(0.05)	(0.04)	(0.06)
Primary	–	Ref.	Ref.	Ref.	Ref.
Tenure					
Private rent	–	0.00	0.00	–	–
		(0.01)	(0.01)		
Reduced rent	–	−0.02	−0.01	–	-
		(0.01)	(0.01)		
Owner occupier	–	Ref.	Ref.	–	–
Disposable income (1000 s)	–	0.00	0.00	0.00	−0.00
		(0.00)	(0.00)	(0.00)	(0.00)
Chronic Illness at baseline year (yes)	–	–	–	–	–
Limiting illness at baseline year					
Yes, strongly limiting	–	–	–	–	–
Yes	–	–	–	–	–
No	–	Ref.	Ref.	Ref.	Ref.
Economic activity					
Lost job	–	0.03**	0.03*	0.02	0.02
		(0.01)	(0.01)	(0.02)	(0.01)
Retired	–	−0.01	−0.01	−0.03	0.02
		(0.03)	(0.03)	(0.02)	(0.05)
Other inactive	–	0.01	0.01	0.02	−0.01
		(0.02)	(0.02)	(0.02)	(0.02)
Employed	–	Ref.	Ref.	Ref.	Ref.
Individual fixed effects	N	N	N	N	N
Country-level variables	N	N	Y	Y	Y
Number of individual-years	101483	99749	91547	65264	26285
Countries in sample	27	27	24	24	24

Note: Renters include those who rent at a reduced rate and market rate. Country-level variables are those population level variables given in Table 2. Good/very good health coded as 0, fair/bad/very bad as 1. Fixed effects with clustered standard errors.

* ^*p* <0 .05.^

** ^*p* < 0.01.^

*** ^*p* <0 .001.^

**Table A3:** Sample descriptives disaggregated by tenure.

	Owners	Renters	Statistical test (difference)
Housing payment arrears	1.95%	4.09%	*p* <0 .05
Age	43.04 (S.D. 10.70)	40.39 (S.D. 11.74)	*p* < 0.05
Marital status	Married 69.58%	45.80%	*p* < 0.05
	Single 22.51%	39.62%	
	Divorced 6.65%	12.94%	
	Widowed 1.26%	1.64%	
Education	Primary 3.84%	9.93%	*p* < 0.05
	Secondary 56.43%	61.44%	
	Post-secondary 39.73%	28.63%	
Disposable income	47665.13 (36255.76)	33083.15 (25852.57)	*p* < 0.05
Chronic illness	19.60%	20.12%	*p* < 0.05
Limiting illness	No 88.29%	86.04%	*p* < 0.05
	Yes 9.76%	11.38%	
	Strongly limiting 1.95%	2.58%	
Economic activity	Employed 94.54%	91.80%	*p* < 0.05
	Unemployed 1.99%	4.01%	
	Retired 1.52%	1.62%	
	Inactive 1.96%	2.57%	

## Appendix A

See Table A1, Table A2, Table A3.
